# A large deletion in the *COL2A1* gene expands the spectrum of pathogenic variants causing bulldog calf syndrome in cattle

**DOI:** 10.1186/s13028-020-00548-w

**Published:** 2020-09-07

**Authors:** Joana Gonçalves Pontes Jacinto, Irene Monika Häfliger, Anna Letko, Cord Drögemüller, Jørgen Steen Agerholm

**Affiliations:** 1 Department of Veterinary Medical Sciences, University of Bologna, Via Tolara di Sopra 50, Ozzano dell’Emilia, 40064 Bologna, Italy; 2 Institute of Genetics, Vetsuisse Faculty, University of Bern, Bremgartenstr. 109a, 3001 Bern, Switzerland; 3 Department of Veterinary Clinical Sciences, University of Copenhagen, Højbakkegaard Allé 5A, 2630 Taastrup, Denmark

**Keywords:** Chondrodysplasia, Congenital, Malformation, Precision medicine, Rare disease, Type II collagenopathy, Whole-genome sequencing

## Abstract

**Background:**

Congenital bovine chondrodysplasia, also known as bulldog calf syndrome, is characterized by disproportionate growth of bones resulting in a shortened and compressed body, mainly due to reduced length of the spine and the long bones of the limbs. In addition, severe facial dysmorphisms including palatoschisis and shortening of the viscerocranium are present. Abnormalities in the gene *collagen type II alpha 1 chain* (*COL2A1*) have been associated with some cases of the bulldog calf syndrome. Until now, six pathogenic single-nucleotide variants have been found in *COL2A1*. Here we present a novel variant in *COL2A1* of a Holstein calf and provide an overview of the phenotypic and allelic heterogeneity of the *COL2A1*-related bulldog calf syndrome in cattle.

**Case presentation:**

The calf was aborted at gestation day 264 and showed generalized disproportionate dwarfism, with a shortened compressed body and limbs, and dysplasia of the viscerocranium; a phenotype resembling bulldog calf syndrome due to an abnormality in *COL2A1*. Whole-genome sequence (WGS) data was obtained and revealed a heterozygous 3513 base pair deletion encompassing 10 of the 54 coding exons of *COL2A1*. Polymerase chain reaction analysis and Sanger sequencing confirmed the breakpoints of the deletion and its absence in the genomes of both parents.

**Conclusions:**

The pathological and genetic findings were consistent with a case of “bulldog calf syndrome”. The identified variant causing the syndrome was the result of a de novo mutation event that either occurred post-zygotically in the developing embryo or was inherited because of low-level mosaicism in one of the parents. The identified loss-of-function variant is pathogenic due to *COL2A1* haploinsufficiency and represents the first structural variant causing bulldog calf syndrome in cattle. Furthermore, this case report highlights the utility of WGS-based precise diagnostics for understanding congenital disorders in cattle and the need for continued surveillance for genetic disorders in cattle.

## Background

The bulldog calf syndrome (BDS) is a congenital form of bovine chondrodysplasia affecting bones with endochondral osteogenesis. In its most severe form, this syndrome is lethal [1]. The BDS is often exemplified by the Dexter BDS type [2], which is linked to abnormalities in the *aggrecan (ACAN*) gene [3]. However other BDS types, which share gross morphology features with Dexter type, are associated with abnormalities in other genes and occur in different cattle breeds. Abnormalities in the *collagen type II alpha 1 chain* (*COL2A1*) gene causing BDS have been reported several times during the last 15 years (achondrogenesis/hypochondrogenesis type II in *Bos taurus*; OMIA 001926-9913; https://omia.org/OMIA001926/9913/). The purpose of this study was to report a variant in the *COL2A1* gene leading to BDS and provide an overview of the phenotypic and allelic heterogeneity of *COL2A1*-related BDS.

## Case presentation

A stillborn Holstein male calf with a body weight of 18.1 kg was aborted at gestation day 264 (normal gestation 281 days (mean)). The pregnancy was the result of insemination with semen of a purebred Holstein sire on a Holstein dam. The parents were not related within at least four generations. The calf had moderate autolysis and was frozen at − 20 °C before submission for necropsy and was examined after thawing.

**Fig. 1:**
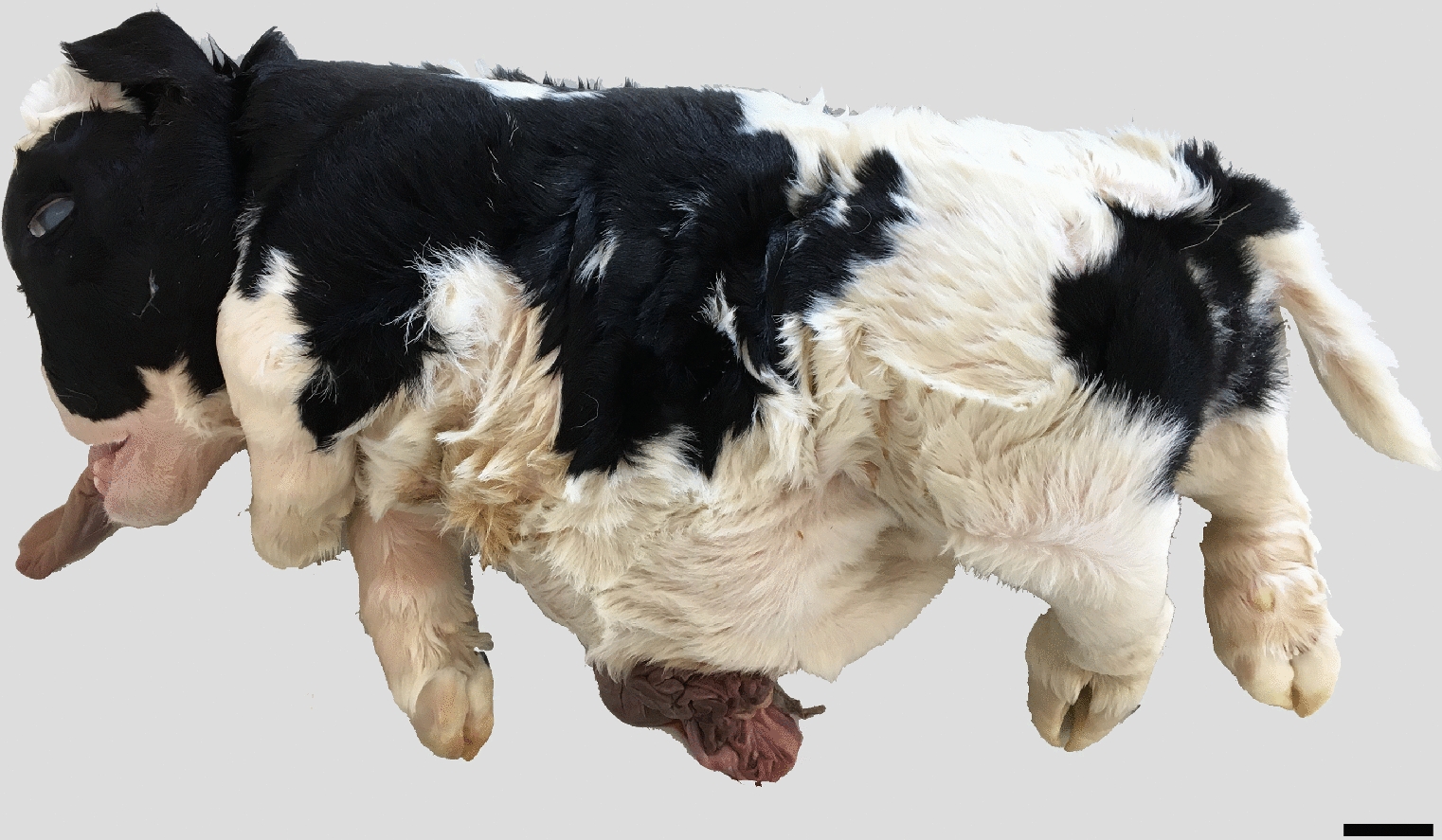
Calf affected by the OMIA 001926-9913 bulldog calf syndrome. Note the short compact body and limbs and the malformed head. Segments of the intestine protruded through a defect in the ventral abdominal midline, probably as a result of insufficient space within the abdomen due to the short spine. Bar: 5 cm

**Fig. 2:**
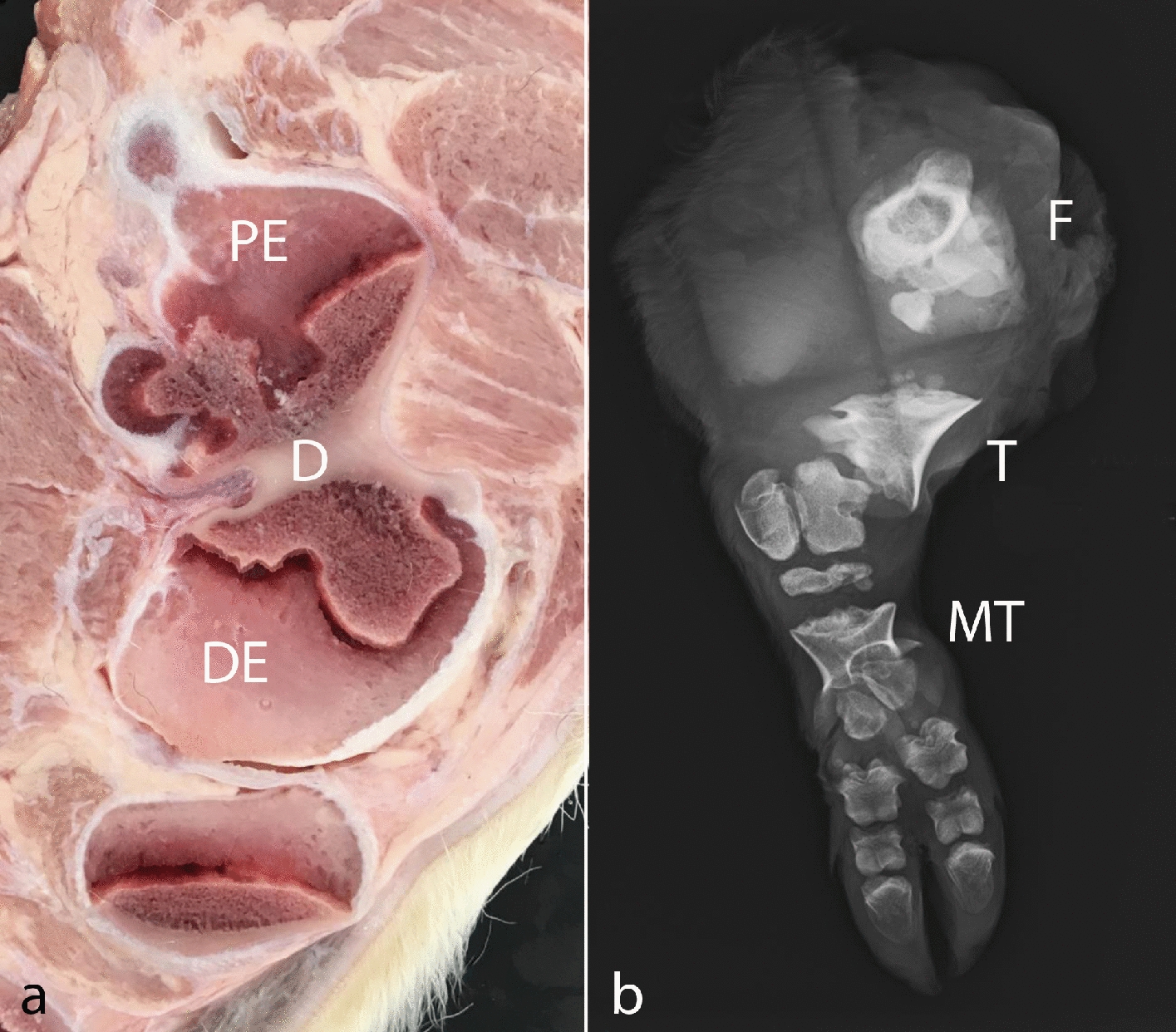
Lesions in the bones of the limbs. **a** Parasagittal longitudinal section through the femur showing a dysplastic irregular shaped diaphysis (D) bordered by enlarged chondroid proximal and distal epiphyses (PE and DE, respectively). **b** Radiographic image of a hind limb illustrating the severe bone abnormalities of most bones. Of the femur (F), tibia (T) and metatarsal bones (MT), only their irregularly shaped diaphysis are seen and these bones were only identifiable by their location. The phalangeal bones were well developed and of normal shape

**Fig. 3:**
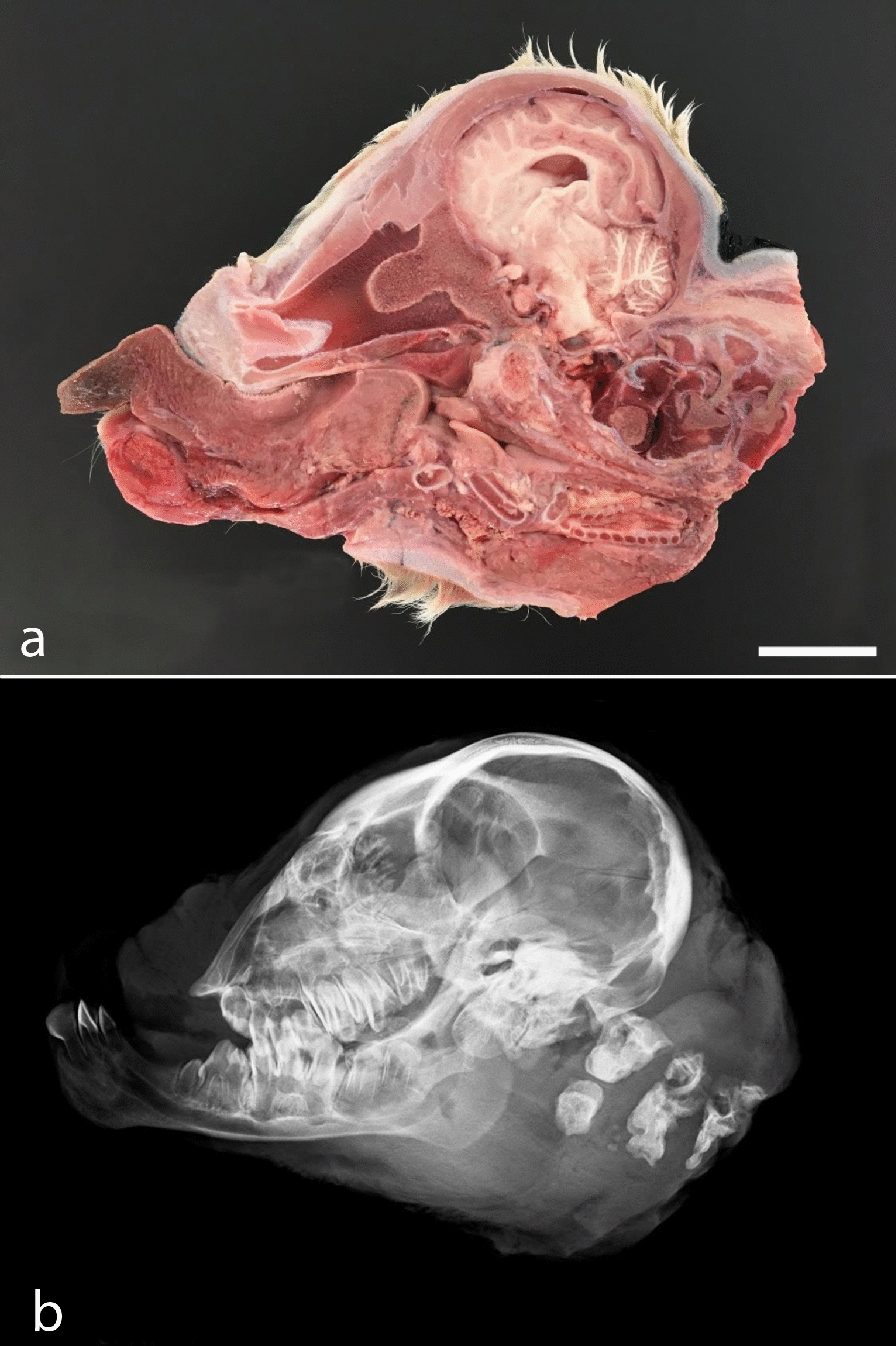
Gross morphology and radiographic findings in the head. **a** Midline section showing the shortened dysplastic face and the dorso-caudal angling of the brain stem (indicated by black lines). The section through the spine is slightly parasagttital, so the spinal cord is not seen. Frozen specimen. Bar: 5 cm. **b** Same specimen as in **a** highlightening the abnormally shaped bones

The calf had generalized disproportionate dwarfism resembling a case of BDS (Fig. 1). The body appeared shortened and compact. The limbs showed bilateral symmetric shortening, which especially affected the bones proximal to the phalanges, giving the limbs a compact appearance. The phalanges were slightly rotated medially. The limbs were sawed longitudinally, which confirmed the irregular development of diaphysis and the presence of enlarged chondroid epiphyses without ossification centers (Fig. 2a). Radiological examination prior to sawing revealed normally structured phalangeal bones, but otherwise bones were only seen as irregular diaphyseal segments that could only be identified based on their location (Fig. 2b). Vertebrae had a similar appearance with enlarged chondroid epiphyses and irregular diaphyses. The head had dysplasia of the viscerocranium with shortening of the maxillary bones, palatoschisis, protrusion of the tongue and doming of the calvarium (Fig. 3). Longitudinal sawing of the head through the midline revealed that the direction of the brain axis was elevated due to the abnormally shaped neurocranium (Fig. 3a). Radiological examination highlighted the abnormally shaped bones (Fig. 3b). The thorax was narrow and of reduced volume and was mostly occupied by an enlarged malformed heart. The heart malformation consisted of bilateral ventricular dilation, muscular hypertrophy of the right ventricular wall and dilation of the pulmonary trunk. The lung was hypoplastic and nonaerated. The abdomen was dilated with eventration of intestinal segments and the liver appeared indurated. Due to the level of autolysis and freezing artefacts, histopathology was not performed.

### Genetic analysis

**Fig. 4:**
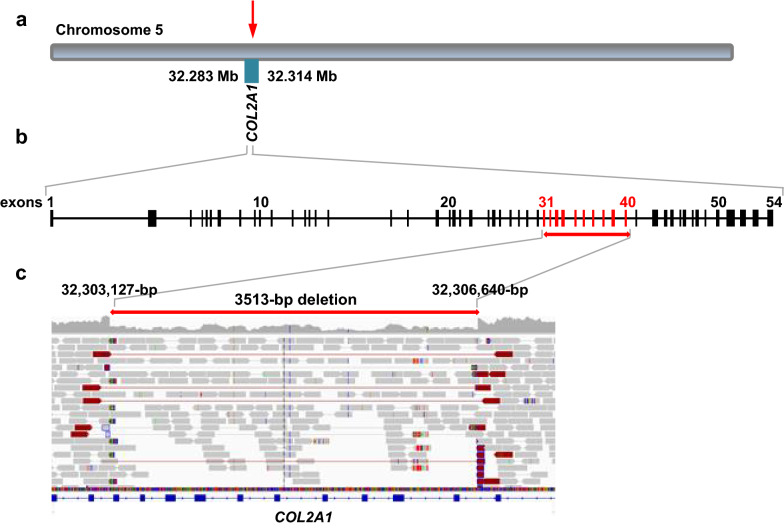
Schematic diagram of the *COL2A1* gene showing the pathogenic variant causing BDS in the affected calf. **a** Location of the bovine *COL2A1* gene and causal variant indicated by the arrow on chromosome 5 of the ARS-UCD1.2 bovine genome assembly. **b** Schematic illustration of the bovine *COL2A1* gene with its 54 annotated exons. **c** Integrative Genomics Viewer screenshot in the region of the heterozygous 3513‐bp deletion in combination affecting exons 31 to 40 of the *COL2A1* gene. Note the drop in coverage and the truncated read‐alignments at the deletion breakpoints. Sequence reads coloured in dark red indicate the deletion as read‐pairs that map farther apart on the reference genome than the average insert size of the sequencing library

Whole-genome sequencing using the NovaSeq 6000 (illumina) was performed at a read depth of ~ 26× using DNA extracted from skin and cartilage from the ear of the calf. The generated sequences were mapped to the ARS‐UCD1.2 reference genome, and single-nucleotide variants (SNVs) and small indel variants were called. The applied software and steps to process fastq files into binary alignment map (BAM) and genomic variant call format (GVCF) files were in accordance with the latest 1000 Bull Genomes Project processing guidelines (www.1000bullgenomes.com) [4]. Furthermore, CombineGVCFs and CatVariants of GATK v3.8 [5] were used to combine the GVCF files and the VariantFiltration tool of GATK was used to give the variants quality labels based on the standard GATK best practices. Lastly, functional impacts were annotated using SNPEFF v4.3 [6] by integrating the information from the NCBI Annotation Release 106 (https://www.ncbi.nlm.nih.gov/genome/annotation_euk/Bos_taurus/106/). With the resulting GVCF, including all individual variants and their functional predictions, filtering for private variants was performed. We compared the genotypes of the calf with 494 cattle genomes of various breeds that had been sequenced in the course of other ongoing studies. The WGS data of the case can be found on ENA under the sample accession number SAMEA6528902, while a comprehensive list with all ENA accession numbers is shown in Additional file 1. A total of 20 private protein-changing single-nucleotide or short indel variants with a moderate or high predicted impact, located within 19 different genes or loci, were identified (Additional file 2). This list included no variants in *COL2A1*, the most likely candidate gene for the observed BDS phenotype. Therefore, Integrative Genomics Viewer (IGV) [7] software was used for visual inspection of the genome region containing *COL2A1* on chromosome 5. A heterozygous 3513 base pair (bp) sized deletion from position 32,303,127 to 32,306,640 spanning 10 coding exons of the *COL2A1* gene leading to haploinsufficiency of the encoded collagen type II alpha 1 chain protein was observed (Fig. 4). The heterozygous gross deletion variant in *COL2A1* is predicted to lead to a loss-of-function of the encoded collagen type II alpha 1 chain protein and was not observed in any of the 494 cattle genomes used for comparison. Therefore, this variant was further investigated as a potentially causative variant for the observed phenotype.

To evaluate whether the deletion in *COL2A1* occurred de novo, the affected genomic region was amplified by polymerase chain reaction (PCR) and Sanger sequenced using DNA of the calf and both parents. Genomic DNA was extracted from EDTA blood and semen of the dam and sire respectively, and compared to the calf’s DNA. PCR products were obtained by using primers flanking the detected *COL2A1* deletion (forward: 5′-CAGGGGATGGGTCTTCCT-3′ and reverse: 5′-GCGTTAGAGAGGGAGACAGG-3′) and subsequently sequenced on an ABI3730 capillary sequencer (Thermofisher, Darmstadt, Germany). Only with the DNA of the affected calf, a PCR product of 128 bp could be amplified, whereas for both parents the amplification failed. Sanger sequencing of the obtained amplicon confirmed the previously identified breakpoints in combination with the insertion of a 10-bp segment fused in-between (chr5:g.32303127_32306640delinsTCTGGGGAGC).

## Discussion and conclusions

**Fig. 5:**
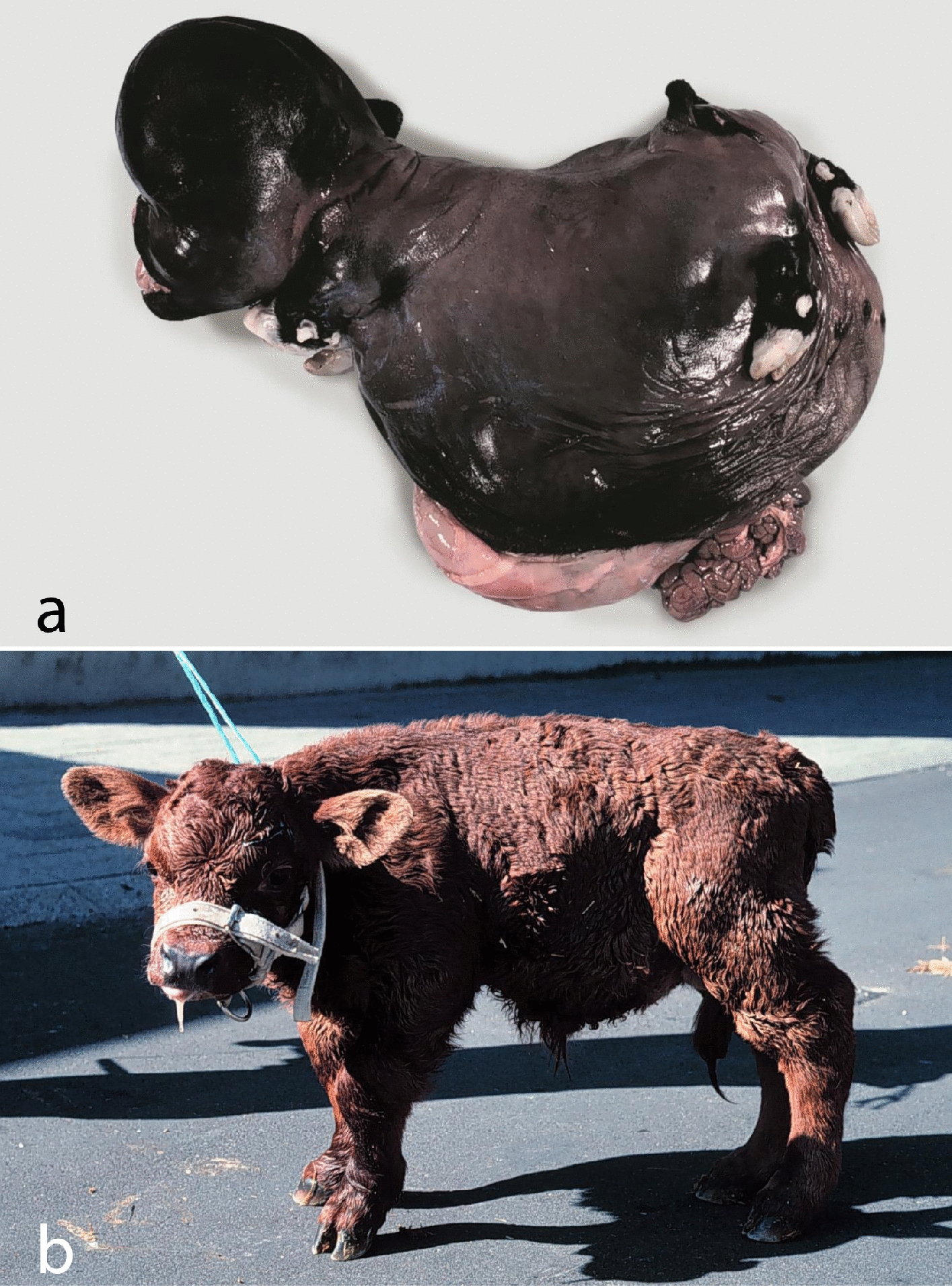
Examples of the bulldog calf syndrome showing the wide spectrum of phenotypes. **a** The bulldog calf syndrome “prototype” as it occurs in Dexter cattle due to mutations in the *ACAN* gene. Aborted Dexter fetus; **b** Autosomal recessively inherited sublethal form of the bulldog calf syndrome as it occurred in Red Danish cattle [15]. The molecular basis of this phenotype is unknown

Based on the morphology of the presented BDS case, a causative genetic variation in the *COL2A1* gene was suspected. As for humans, the morphology of BDS in cattle vary widely both in the overall gross morphology and in bone morphology as exemplified in Fig. 5. It appears that cases of BDS due to abnormalities in the *COL2A1* gene share a common morphology that separates them from at least some other types of bovine BDS, although few types of bovine BDS have been characterised to the molecular level. BDS cases due to abnormalities in the *COL2A1* gene are delivered at term or during the last 3 weeks of gestation. The affected calves have a significantly reduced body weight with a mean of 22.3 kg (variation 16.3–27.5 kg) for 11 Holstein cases [1, 8, 9]. The body and limbs are short and compressed with the digits being almost half of normal size, but normally shaped. The long bones of the limbs and the vertebrae have small irregular diaphyses and enlarged chondroid epiphyses. The viscerocranium is dysplastic with palatoschisis, the neurocranium doomed causing dorso-caudal rotation of the brain, the heart is malformed due to the narrow-spaced thorax, the lungs compressed and the liver with signs of chronic stasis. Cases of BDS that share this morphology, should be suspected of having a defect in the *COL2A1* gene; a suspicion that is helpful when analysing WGS data. However, in this case, filtering for private variants in *COL2A1* did not lead to the detection of a private single-nucleotide or short indel variant. Consequently, the genome data was visually inspected for the presence of structural variants in the gene that allowed the detection of a heterozygous gross deletion. It was assumed that it had occurred either post-zygotically in the developing embryo or was inherited from a parent having low-level mosaicism. The former seems to be more likely as amplification of the mutant allele failed in the examined tissues of both parents, especially because the germline of the sire was analysed by extracting DNA from semen. This means that the *COL2A1* deletion observed in heterozygous state in the affected offspring was most likely absent in the genome of both parents. Therefore, we can assume that the identified mutation arose indeed de novo in the developing embryo explaining this isolated case.

**Fig. 6:**
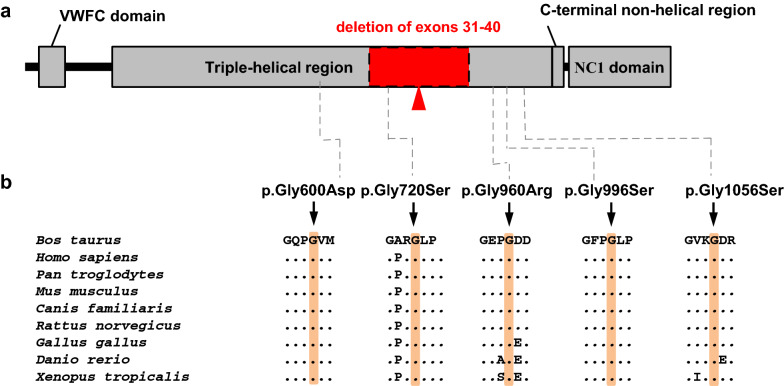
Schematic diagram of the COL2A1 protein. **a** Domain and region information for the α1 chain of type II collagen (COL2A1) obtained from the UniProt database (http://www.uniprot.org/; accession number: P02459). Note that the large deletion (red arrow) affects the triple-helical region. **b** Multiple species alignment of the COL2A1 proteins across 9 different species showing a complete conservation of the affected residue. Note the pathogenic exchange of glycine residues of the Gly-x-y structural motif which is typical of triple-helical regions from collagen proteins reported in BDs-affected cases: p.Gly600Asp in Charolais Salers crossbreed calves, p.Gly720Ser in an isolated German Holstein calf [5], p.Gly960Arg in French Holstein calves [13], p.Gly996Ser in German Holstein calves [6] and p.Gly1056Ser in an isolated BD Holstein calf [14]. Protein sequences accession numbers in NCBI for each species are NP_001001135.2 (*Bos taurus*), NP_001835.3 (*Homo sapiens*), XP_509026.2 (*Pan troglodytes*), NP_112440.2 (*Mus musculus*), NP_001006952.1 (*Canis familiaris*), NP_037061.1 (*Rattus norvegicus*), NP_989757.1 (*Gallus gallus*), NP_571367.1 (*Danio rerio*) and NP_989220.1 (*Xenopus tropicalis*)

This pathogenic variant is predicted to affect a large portion of the *COL2A1* gene leading to haploinsufficiency. Recent large data from human genome sequencing studies presented in the Genome Aggregation Database (gnomAD) [10] showed that the probability of loss-of-function intolerance score for *COL2A1* was 1 meaning that *COL2A1* falls into the class of loss-of-function haploinsufficient genes. Collagens are normally extracellular structural proteins involved in formation of connective tissue structure. The highly conserved sequence predominantly consists of repeated three amino acids with glycine (Gly) followed by two other amino acids (Gly-x-y, where x and y can be any amino acid) but glycine being mandatory for the tight packing of the polyproline II type helices within the triple helix [11] (Fig. 6). We assume that the pathogenic variant reported in this study disrupted the triple-helical region of alpha 1 (II) chain and caused a dominant-negative effect similar to most of the alterations responsible for achondrogenesis/hypochondrogenesis type II (OMIM 200610; https://www.omim.org/entry/200610) in human patients. In man, variants in *COL2A1* are associated with 15 different phenotypes exclusively following dominant inheritance (OMIM 120140).

Interestingly, the OMIA 001926-9913 BDS type occurs either as de novo or inherited from a mosaic parent [12]. Mosaic sires have been found to transmit the dominant genetic abnormality to their offspring at rates ranging from 1 to 21% [12, 13] reflecting at what fetal developmental stage the gene change occurred. As it cannot be predicted if the abnormality is occurring de novo or if it is transmitted from a parent, cases must be analyzed in detail to prevent the birth of large numbers of defective offspring, in particular if the abnormality is transmitted from breeding sire with high generic merit used for artificial breeding.

A total of six independent pathogenic dominant variants in *COL2A1*, considered to be responsible for BDS, have been previously identified [8, 9, 12–14] (Table 1). All these variants involve a single nucleotide; five out of the six reported variants represent missense variants that cause a change in a glycine residue disrupting the Gly-x–y structural motif essential for the assembly of the collagen triple-helix.

**Table 1:** Previously reported genetic variants of *COL2A1* causing the OMIA 001926-9913 bulldog calf syndrome in cattle

Inheritance	Type of variant	Variant^a^	Breed	References
AD, mosaicism	Missense	g.32307658G > A p.Gly960Arg	Holstein	[13]
AD, mosaicism	Splicing	g.32305226G > A	Holstein	[8]
AD, mosaicism	Missense	g.32301746G > A p.Gly600Asp	Charolais × Salers	[12]
AD, de novo	Missense	g.32303739G > A p.Gly720Ser	Holstein	[12]
AD, mosaicism	Missense	g.32308008G > A p.Gly996Ser	Holstein	[9]
AD, de novo	Missense	g.32308734G > A p.Gly1056Ser	Holstein	[14]

*AD* autosomal dominant, *OMIA* Online Mendelian Inheritance in Animals, https://omia.org/home/

^a^Given positions correspond to chromosome 5 of the ARS-UCD1.2 assembly and NP_001001135.2

This is the first report of a large deletion in the *COL2A1* gene associated with BDS. The previous reported single nucleotide variants were missense and splicing. The relevance of this case report is to show that also larger-sized genomic deletions cause a similar congenital phenotype and thereby expanding the knowledge on this condition by emphasizing that different mutations in *COL2A1* cause a uniform phenotype. For many genes it is known that the kind of genetic alteration influence the phenotypic outcome, e.g. the severity of a congenital defect varies or differs totally depending on the individual variant. Interestingly for *COL2A1* in cattle this seems not to be the case as different kinds of variants always cause an identical phenotype which is of importance for diagnostic pathologists. Furthermore, this report provides an overview of the phenotypic and allelic heterogeneity of the *COL2A1*-related BDS in cattle. This example highlights the utility of WGS-based precise diagnostics for understanding disorders linked to de novo mutations in animals with an available reference genome sequence and the need for continued surveillance for genetic disorders in cattle breeding. Genome sequencing might improve the precision of the clinicopathological diagnosis as sometimes unexpected variants in genes that were not known to be associated with a certain disorder could be detected.

## Supplementary information

**Additional file 1.** EBI Accession numbers of all publicly available genome sequences. We compared the genotypes of the calf with 494 cattle genomes of various breeds that had been sequenced in the course of other ongoing studies and that were publicly available.

**Additional file 2.** List of the remaining private protein-coding variants after comparison of the genotypes of the calf with 494 cattle genome. A total of 20 private protein-changing single-nucleotide or short indel variants with a moderate or high predicted impact, located within 19 different genes or loci, were identified.

## Data Availability

Whole-genome sequence data generated from the affected calf is available under study accession PRJEB18113 and sample accession SAMEA6528902 from the European Nucleotide Archive (ENA). In addition, further control genomes are listed in Additional file 1 and can also be accessed on ENA.

## Abbreviations

BAM: Binary alignment map
BDS: Bulldog calf syndrome
Bp: Base pair
*COL2A1*: Collagen type II alpha 1 chain
EDTA: Ethylenediaminetetraacetic acid
Gly: Glycine
gnomAD: Genome Aggregation Database
GVCF: Genomic variant call format
IGV: Integrative Genomics Viewer
OMIA: Online Mendelian Inheritance in Animals, https://omia.org/home/
OMIM: Online Mendelian Inheritance in Man, https://omim.org/
PCR: Polymerase chain reaction
SNV: Single-nucleotide variant
WGS: Whole-genome sequencing
